# Cytochrome P450 26A1 Contributes to the Maintenance of Neuropathic Pain

**DOI:** 10.1007/s12264-023-01101-1

**Published:** 2023-08-28

**Authors:** De-Li Cao, Ling-Jie Ma, Bao-Chun Jiang, Qiang Gu, Yong-Jing Gao

**Affiliations:** 1 Institute of Pain Medicine and Special Environmental Medicine, Co-innovation Center of Neuroregeneration, Nantong University, Nantong, 226019 China; 2 Nantong University Medical School, Nantong, 226001 China; 3 Department of Pain Management, The Affiliated Hospital of Nantong University, Nantong, 226001 China

**Keywords:** CYP26A1, Microglia, Astrocytes, ERK, P38, IL-10, Neuropathic pain

## Abstract

The cytochrome P450 proteins (CYP450s) have been implicated in catalyzing numerous important biological reactions and contribute to a variety of diseases. CYP26A1, a member of the CYP450 family, carries out the oxidative metabolism of retinoic acid (RA), the active metabolite of vitamin A. Here we report that CYP26A1 was dramatically upregulated in the spinal cord after spinal nerve ligation (SNL). CYP26A1 was mainly expressed in spinal neurons and astrocytes. HPLC analysis displayed that the content of all-trans-RA (at-RA), the substrate of CYP26A1, was reduced in the spinal cord on day 7 after SNL. Inhibition of CYP26A1 by siRNA or inhibition of CYP26A1-mediated at-RA catabolism by talarozole relieved the SNL-induced mechanical allodynia during the maintenance phase of neuropathic pain. Talarozole also reduced SNL-induced glial activation and proinflammatory cytokine production but increased anti-inflammatory cytokine (IL-10) production. The RA receptors RARα, RXRβ, and RXRγ were expressed in spinal neurons and glial cells. The promoter of *Il-10* has several binding sites for RA receptors, and at-RA directly increased *Il-10* mRNA expression *in vitro*. Finally, intrathecal IL-10 attenuated SNL-induced neuropathic pain and reduced the activation of astrocytes and microglia. Collectively, the inhibition of CYP26A1-mediated at-RA catabolism alleviates SNL-induced neuropathic pain by promoting the expression of IL-10 and suppressing glial activation. CYP26A1 may be a potential therapeutic target for the treatment of neuropathic pain.

## Introduction

The cytochrome P450 (CYP450) family is a superfamily of heme-binding monooxygenases that catalyze numerous important biological reactions, including the non-specific oxidative conversion of steroids, lipids, xenobiotics, and environmental toxins [1, 2]. The CYP450 superfamily contains >70 families, each of which is constituted by a plurality of family members. These family members are distinct in their particular distribution, biologically active pattern, or preferred substrate [1–3]. Studies have shown that CYP450s are associated with a variety of diseases, such as cancer, liver diseases, inflammatory diseases, and neurodegenerative diseases [4–7].

The CYP26 family - CYP26A1, CYP26B1, and CYP26C1 - carry out oxidative metabolism of retinoic acid (RA), the active metabolite of vitamin A [8, 9]. RA is a powerful regulator of gene transcription and plays a crucial role in cellular proliferation and differentiation. Consistent with this, the CYP26 family is important in regulating neuronal development, differentiation, and survival [10–13]. It has been demonstrated that CYP26A1 is pivotal in embryonic development, whereas CYP26B1 is essential for postnatal survival and germ-cell development [14–19]. In the adult, CYP26A1 is highly expressed in the liver, less in the brain and testis, and CYP26B1 is expressed mainly in brain tissue, but also at low levels in other tissues [20–22]. CYP26C1 is mainly expressed during embryonic development and has a very low level in adult tissues [21, 23, 24]. In contrast to the well-studied role of the CYP26 family in the embryonic central nervous system (CNS), relatively little is known about their functions in adults. A recent study showed that the knockdown of CYP26B1 in the nucleus accumbens shell increases depression-related behavior while decreasing anxiety-like behavior [25], implicating the function of CYP26B1 in the adult CNS.

Neuropathic pain is a direct consequence of a lesion or disease affecting the somatosensory system. Neuroinflammation mediated by neurons and glial cells (astrocytes and microglia) plays a critical role in the pathogenesis of neuropathic pain [26, 27]. In addition, cytokine- and chemokine-mediated interactions between neurons and glial cells modulate glial activation and synaptic plasticity after nerve injury [27, 28]. All-trans-RA (at-RA) reduces the lipopolysaccharide (LPS)-induced production of inflammatory chemokines, cytokines, and PGE2 in astrocytes [29–31]. RA decreases the synthesis of TNF-α and enhances the production of IL-10 from LPS-stimulated monocytes/macrophages [32, 33]. Whether the CYP26 family regulates neuroinflammation in the spinal cord and contributes to neuropathic pain has not been investigated.

In the present study, using the spinal nerve ligation (SNL)-induced neuropathic pain model in mice, we screened the gene expression of CYP450 superfamily members in the spinal cord and found that CYP26A1 is highly upregulated 10 days after SNL. Our results demonstrated that the increased CYP26A1 is associated with decreased at-RA in the spinal cord. In addition, CYP26A1-mediated at-RA degradation contributed to spinal glial activation *via* regulating IL-10 production.

## Materials and Methods

### Animals and Surgery

Adult ICR mice (male, 7–8 weeks old) were purchased from the Experimental Animal Center of Nantong University. The animals were maintained at a room temperature of 23 ± 1°C, a humidity of 55% ± 10%, and a 12:12 light-dark cycle with free access to food and water. All animal procedures were performed according to the guidelines of the International Association for the Study of Pain and were approved by the Animal Care and Use Committee of Nantong University. The L4 SNL was produced as previously described [34]. For the sham operation, the L4 spinal nerve was exposed, but not ligated.

### Drugs and Administration

Talarozole (R115866), a potent and selective inhibitor of cytochrome P450 26-mediated breakdown of at-RA, was purchased from Med Chem Express (New Jersey, USA). At-RA was from Sigma-Aldrich (St. Louis, MO, USA). For intrathecal injection, a spinal cord puncture was made with a 30-G needle between the L5 and L6 levels to deliver the reagents to the cerebral spinal fluid [35].

### Quantitative Real-time PCR (qPCR)

Total RNA was extracted from the L4 spinal cord with the TRIzol reagent (Invitrogen, Carlsbad, CA). RNA (1 µg) was converted into cDNA using a HiScript II 1^st^ Strand cDNA Synthesis Kit (Vazyme, Nanjing, China). The cDNA was amplified using the following primers: *Cyp26a1* forward, 5′-TGC AAG AGC AAT CAA GAC AAC A-3′; C*yp26a1* reverse, 5′-CTT CAG AGC AAC CCG AAA CC-3′; *Cyp26b1* forward, 5′-CTC TGC CCC TTT GCT CTT G-3′; *Cyp26b1* reverse, 5′-TCT TTC CAC CTT ACC TCT CTG CTT-3′; *Gfap* forward, 5′-CCA AGA TGA AAC CAA CCT GA-3′; *Gfap* reverse, 5′-TCC AGC GAT TCA ACC TTT C-3′; *Aif1* (encoding IBA-1) forward, 5′-ATG AGC CAA AGC AGG GAT T-3′; *Aif1* reverse, 5′-CTT CAA GTT TGG ACG GCA G-3′; *Tnf* forward, 5′-GTT CTA TGG CCC AGA CCC TCA C-3′; *Tnf* reverse, 5′-GGC ACC ACT AGT TGG TTG TCT TTG-3′; *Il-1* forward, 5′-TCC AGG ATG AGG ACA TGA GCA C-3′; *Il-1* reverse, 5′-GAA CGT CAC ACA CCA GCA GGT TA-3′; *Il-10* forward, 5′-GAC CAG CTG GAC AAC ATA CTG CTA A-3′; *Il-10* reverse, 5′-GAT AAG GCT TGG CAA CCC AAG TAA-3′; *Gapdh* forward, 5′-GTA AGA AAC CCT GGA CCA CCC-3′; *Gapdh* reverse, 5′-AGG GAG ATG CTC AGT GTT GG-3′. The AceQ qPCR SYBR Green Master Mix (Vazyme) was used for all PCR reactions, which were run on Step One Plus Real-Time PCR instrument (Applied Biosystems, CA, USA). The PCR amplification was performed at 95°C for 3 min, followed by 40 cycles at 95°C for 10 s, and 60°C for 30 s. The melting curves were obtained at 95°C for 15 s, 60°C for 60 s, and 95°C for 15 s to validate the utility and specificity of each PCR product. The data were analyzed using the Comparative CT Method (2^−ΔΔCT^).

### *In situ* RNA Hybridization

The cellular localization of *Cyp26a1* mRNA was determined with an RNAscope 2.5 HD Detection Kit (RED) containing the probe for mouse *Cyp26a1* mRNA (Advanced Cell Diagnostics, Inc. Newark, CA, USA) according to the manufacturer’s protocol. Spinal cord cryosections (14 μm) were mounted onto SuperFrost Plus slides (Thermo Scientific) and dried for 1 h at 60°C, followed by washing for 5 min with 0.01 mol/L PBS. After pretreatment with hydrogen peroxide for 5 min at room temperature, sections were washed twice for 2 min with distilled water. The slides were immersed in 100% ethanol for 3 min and dried at room temperature. They were then subjected to protease-plus digestion for 10 min at 40°C, followed by washing twice for 2 min with distilled water. Hybridization was applied with a hybridization probe specific to *Cyp26a1* mRNA or negative control probe in an HybEZ oven (ACD) at 40°C for 2 h, followed by signal amplification steps. The hybridization signal was detected using a mixture of Fast-RED solutions A and B (60:1). The sections were examined under a Leica TCS SP8 confocal laser scanning microscope (Leica, Wetzlar, Germany).

### Immunohistochemistry

After appropriate survival times, animals were deeply anesthetized with isoflurane and perfused through the ascending aorta with PBS followed by 4% paraformaldehyde in 0.1 mol/L PB. After perfusion, the L4 spinal cord segments were removed and postfixed overnight. Spinal cord sections (30 μm) were cut on a cryostat and processed for immunofluorescence as we described previously [36]. The sections were first blocked with 1% BSA with 0.1% Triton X-100 in 0.01 mol/L PBS for 2 h at room temperature. The sections were then incubated overnight at 4°C with the following primary antibodies: CYP26A1 antibody (rabbit, 1:200; Bioss, Beijing, China), c-Fos antibody (Guinea pig, 1:2000; Synaptic Systems, Göttingen, Germany), GFAP antibody (mouse, 1:6000; Millipore, Billerica, MA), IBA-1 antibody (goat, 1:500; Abcam, Cambridge, UK), neuronal-specific nuclear protein (NeuN) antibody (mouse, 1:800; Millipore), RARα antibody (rabbit, 1:500; Bioss), RXRβ antibody (rabbit, 1:500; Bioss), and RXRγ antibody (rabbit, 1:500; Bioss). The sections were then incubated for 2 h at room temperature with Cy3- or Alex 488-conjugated secondary antibodies (1:1000, Jackson ImmunoResearch, Westgrove, PA). The stained sections were examined under a Leica fluorescence microscope, and images were captured with a CCD spot camera.

### Western Blot

Animals were transcardially perfused with PBS 7 days after SNL or sham operation. The lumbar spinal cord segment (L4) was dissected out and the tissue was homogenized in a lysis buffer. Protein concentrations were determined by BCA Protein Assay (Thermal Fisher, Rockford, IL). Thirty micrograms of proteins were loaded and separated on an SDS-PAGE gel. After transfer, the blots were incubated overnight at 4°C with antibodies against CYP26A1 (rabbit, 1:1000; Thermal Fisher, Rockford, IL), pERK (rabbit, 1:1000; CST), pp38 (rabbit, 1:1000; CST), GFAP antibody (mouse, 1:6000; Millipore), and IBA-1 (goat, 1:1000; Abcam). For loading control, the blots were incubated with antibodies against GAPDH (mouse, 1:20000; Sigma-Aldrich), ERK (rabbit, 1:1000; CST), or p38 (rabbit, 1:1000; CST). These blots were further incubated with IRDye 800 CW goat anti-rabbit IgG (H + L) or IRDye 800 CW goat anti-mouse IgG (H + L) for 2 h at room temperature and then examined with the LI-COR Odyssey CLx Imaging System. Specific bands were evaluated by apparent molecular size. The intensity of the selected bands was analyzed using ImageJ software (NIH, Bethesda, MD, USA).

### HPLC Analysis of Endogenous at-RA

The L4 tissue from 4 mice was first homogenized in 30 μL of pre-cooled saline by mortar and pestle on ice. Then 60 μL of acetonitrile (containing 1% glacial acetic acid) was added to the tissue homogenate, shaken for 30 s, and centrifuged at 10,000 g for 10 min at 4°C. The supernatant was pipetted into a brown vial for testing. All laboratory manipulations involving at-RA were performed in a darkened room under dim yellow light to avoid RA isomerization. For the detection and data evaluation of at-RA, the method was adapted from Meyer et al. [37]. A mixture of 86% acetonitrile and 14% water (containing 0.1% glacial acetic acid) was used as the mobile phase. The flow rate was adjusted to 1 mL/min and the column temperature was 25°C. The detection wavelength was set at 350 nm, and the concentration of at-RA was calculated by the external standard method. The At-RA, purchased from Sigma-Aldrich (Missouri, USA), was dissolved in acetonitrile and used to prepare the standard solutions. The standard curve for at-RA established a linear working range of 25 ng/mL–1000 ng/mL.

### Primary Astrocyte Culture

Primary astrocytes were prepared from the cerebral cortex of 2-day-old ICR mice as we described previously [38]. The cerebral cortex was isolated and the meninges were removed in ice-cold Hank’s buffer. The tissue was then minced into ~1 mm pieces, triturated, filtered through a 100-μm nylon mesh (Biosharp Life Sciences, China), and centrifuged at 3000 g for 5 min. The cell pellets were broken with a pipette and resuspended in low-glucose DMEM (Gibco, Grand Island, NY, USA) with 10% fetal bovine serum (FBS) (Gibco). After trituration, the cell suspensions were filtered through a 10-μm screen, plated into six-well culture plates, and cultured for ~10 days. The medium was changed every third day. Once the cells had grown to ~90% confluence, dibutyryl cAMP (150 μmol/L) (Sigma-Aldrich, MO, USA) was added and left for 3 days to induce differentiation. The *Cyp26a1* siRNAs (1 μg) and NC siRNA (RiboBio, Guangzhou, China) were transfected with Lipofectamine 2000 reagent (Invitrogen, CA, USA) for 24 h before LPS (0.1 μg/mL) (Sigma-Aldrich) exposure for 6 h. The sequences of the three *Cyp26a1* siRNAs were as follows: 5′-GCA GCG AAA GAA GGU GAU U-3′ (siRNA-1); 5′-GCA GGA AAU ACG GCU UCA U-3′ (siRNA-2); 5′-GCA AGA GCA AUC AAG ACA A-3′ (siRNA-3). The sequence of the NC siRNA was 5′-GGC UCU AGA AAA GCC UAU GC-3′. After this, the astrocytes were collected for quantitative real-time PCR to analyze the efficiency of RNA interference.

### BV2 Cell Culture

BV2 cells, a microglial cell line, were purchased from Shanghai Institutes for Biological Sciences, Chinese Academy of Sciences. The cells were cultured in six-well plates in high glucose DMEM supplemented with 10% FBS. The cells were incubated with at*-*RA (0.1 μmol/L or 1 μmol/L) or vehicle for 12 h before LPS (0.1 μg/mL) exposure for 6 h. After this, the BV2 cells were collected for quantitative real-time PCR.

#### Dual-Luciferase Reporter Assays

The firefly luciferase reporter plasmids, pGL3-Il10-Promoter, and pGL3-Basic were constructed by Sangon Biotech Co., Ltd. (Shanghai, China). BV2 cells were seeded in 12-well plates. The pGL3-Il10-Promoter or pGL3-Basic firefly luciferase plasmids (500 ng) were transfected into BV2 cells accompanied by pRL-TK *Renilla* luciferase reference plasmids (10 ng) using Lipofectamine 2000 (Invitrogen). After transfection for 24 h, the cells were incubated with at-RA (0.1 μmol/L) for 12 h. After the incubation, the activities of firefly and *Renilla* luciferase were measured using the Dual-Glo Luciferases Assay System (Promega, Madison, WI, USA).

#### Behavioral Analysis

Animals were habituated to the testing environment daily for at least two days before baseline testing. To assess mechanical allodynia after intrathecal injections of inhibitor or siRNA, animals were put in individual transparent plastic chambers on an elevated wire mesh floor. After 30 min habituation, von Frey filaments (Stoelting, Wood Dale, IL) were applied perpendicular to the plantar surface of each hind paw, starting with the 0.6 g filament. The response to the filament was considered positive if immediate flinching, licking/biting, or rapid withdrawal of the stimulated paw was observed. The 50% paw withdrawal threshold was determined by Dixon’s up-down method [39]. Heat hyperalgesia was measured by radiant heat using the Hargreaves apparatus (IITC Life Science Inc.), which measures withdrawal latency of the paw from a noxious radiant heat source with baseline latencies of 10–14 s and a cutoff time of 18 s to prevent tissue injury.

#### Quantification and Statistics

All data are expressed as the mean ± SEM. The behavioral data were analyzed using a two-way analysis of variance (ANOVA) followed by Bonferroni’s test as the multiple comparison analysis. The qPCR and Western blot data were analyzed using one-way ANOVA followed by Bonferroni’s test. Differences between the two groups were compared using Student’s *t*-test. The criterion for statistical significance was *P* <0.05.

## Results

### *Cyp26a1* is the Most Upregulated Member of the CYP450 Superfamily in the Spinal Cord after SNL

**Fig. 1:**
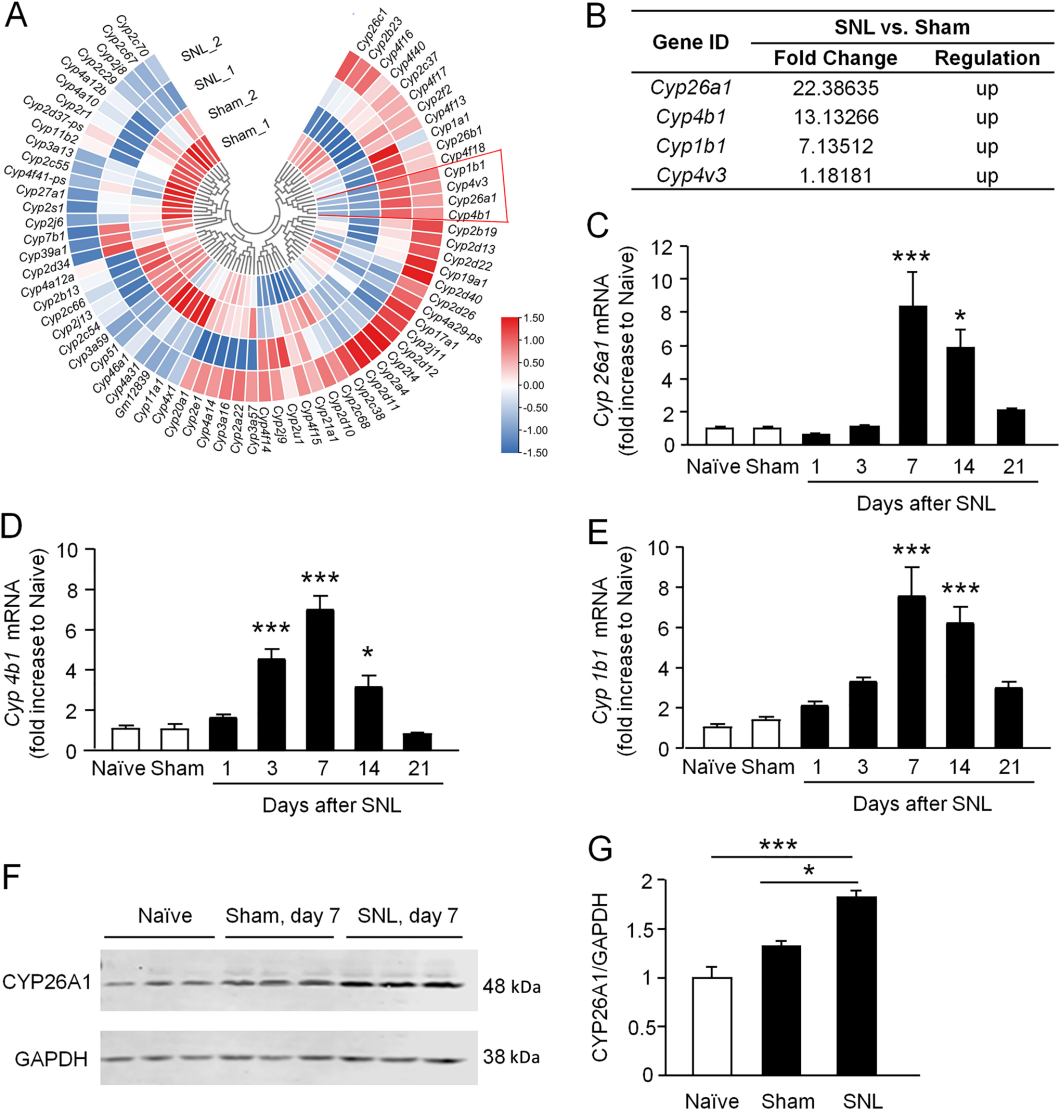
*Cyp26a1* is the most upregulated member of the CYP450 superfamily in the spinal cord in SNL-induced neuropathic pain. **A** Cluster heatmap showing the mRNA expression of 79 cytochrome P450 (CYP) family genes in the L4 spinal cord of SNL and sham-operated mice. The levels of mRNA expression are represented on a logarithmic scale; red corresponds to high expression and blue to low expression. The color scale is shown in the bottom-right corner. **B** Fold change of the four upregulated CYP family genes in the spinal cord after SNL. **C** qPCR showing the time course of *Cyp26a1* mRNA expression in the spinal cord after SNL. *P <0.05, ***P <0.001, SNL *vs* Sham, one-way ANOVA followed by Bonferroni’s test. *n* = 5–6 mice/group. **D, E** qPCR showing the time course of *Cyp4b1* (**D**) and *Cyp1b1* (**E**) mRNA expression in the spinal cord after SNL. *P <0.05, ***P <0.001, SNL *vs* Sham, one-way ANOVA followed by Bonferroni’s test. *n* = 5–6 mice/group. **F, G** Western blots show that spinal CYP26A1 protein expression is increased at 7 days after SNL. *P <0.05, ***P <0.001, SNL *vs* Sham or Naive, Student’s *t*-test, *n* = 3 mice/group.

To investigate which CYP450 may be involved in SNL-induced neuropathic pain, we analyzed the gene expression of CYP450 family members using our previous array data in which gene expression in the ipsilateral spinal cord 10 days after SNL and sham operation was assessed [38]. The results showed that 79 CYP450 superfamily genes were detected, and 3 genes (*Cyp26a1, Cyp4b1, and Cyp1b1*) were upregulated by >5-fold (Fig. 1A). Among them, *Cyp26a1* was the most dramatically increased gene, with a 22-fold increase (Fig. 1B). We further conducted qPCR to check the time course of *Cyp26a1* mRNA expression in the spinal cord after SNL. *Cyp26a1* mRNA did not change on day 1 and day 3 but was significantly increased on day 7 and day 14 in SNL mice compared with sham-operated mice (*F*_(6, 32)_ = 10.67, *P* <0.001, one-way ANOVA, Fig. 1C). *Cyp4b1* mRNA (Fig. 1D) and *Cyp1b1* mRNA (Fig. 1E) were also upregulated in the spinal cord after SNL (*Cyp4b1: F*_(6, 33)_ = 30.54, *P* <0.001; *Cyp1b1*: *F*_(6, 33)_ = 13.71, *P* <0.001, one-way ANOVA followed by Bonferroni’s test). We focused on CYP26A1 and examined the protein level by Western blot. The results showed a significant increase of CYP26A1 protein in the spinal cord 7 days after SNL (*P* <0.05, Student’s *t*-test, SNL *vs* sham, Fig. 1F, G). These data suggest that spinal CYP26A1 may play a role in the maintenance of neuropathic pain.

### CYP26A1 Is Distributed in Neurons and Astrocytes in the Dorsal Horn

**Fig. 2:**
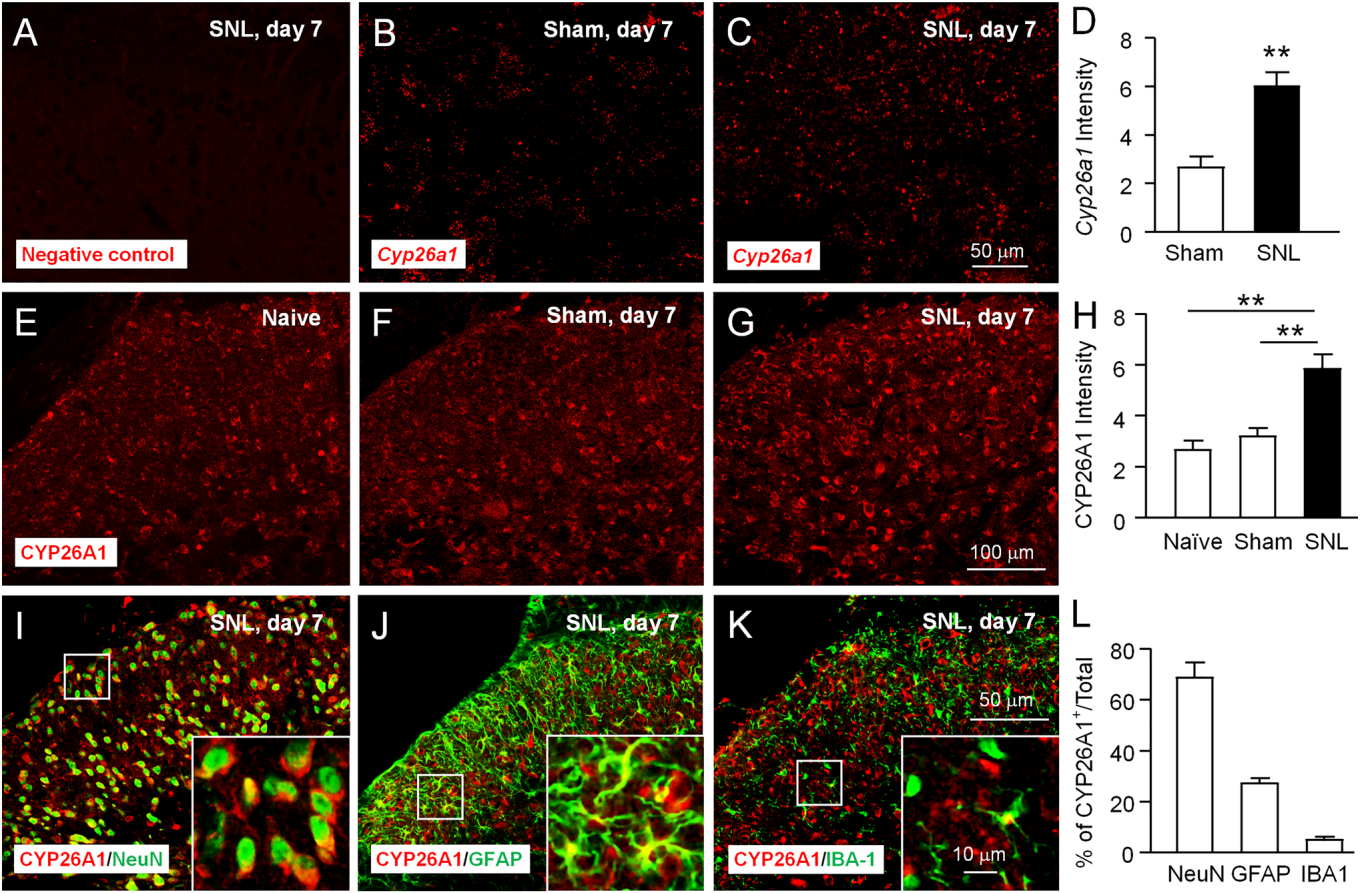
CYP26A1 is expressed in spinal neurons and glial cells after SNL. **A**–**C** Representative images of *in situ* hybridization show the distribution of *Cyp26a1* mRNA in the ipsilateral dorsal horn of sham- and SNL-operated mice. (**A**) is the image from the negative control. **D** Quantification of *Cyp26a1* mRNA intensity. **P <0.01, SNL *vs* Sham, Student’s *t*-test, *n* = 4 mice/group. **E**–**G** Representative images of CYP26A1 immunofluorescence in the ipsilateral dorsal horn. CYP26A1 is constitutively expressed in the spinal dorsal horn in naïve (**E**) and sham-treated mice (**F**) and increased 7 days after SNL (**G**). **H** Quantification of CYP26A1 signal intensity. **P <0.01, SNL *vs* Naïve or Sham, one-way ANOVA followed by Bonferroni’s test, *n* = 4 mice/group. **I**–**K** Double immunofluorescence staining shows that CYP26A1 is mainly colocalized with the neuronal marker NeuN (**I**), partially with the astrocytic marker GFAP (**J**), and a few cells with the microglial marker IBA1 (**K**). **L** The percentage of CYP26A1 in neurons (68.9 ± 5.7%), astrocytes (26.9 ± 2%), and microglia (5.2 ± 1%) of the spinal cord 7 days after SNL. *n* = 4 mice/group. Scale bars, 50 μm (**A–C**), 100 μm (**E–G**), and 50 μm (**I–K**). Scale bar for the inset boxes (**I–K**), 10 μm.

To further investigate the expression and distribution of CYP26A1 mRNA and protein, we applied *in situ* hybridization and immunostaining. *In situ*, hybridization showed that *Cyp26a1* mRNA was increased in the ipsilateral dorsal horn 7 days after SNL (Fig. 2A–D). Immunofluorescence staining also shows that the expression of CYP26A1 was low in naive and sham-operated mice, and increased after SNL (Fig. 2E–H). We then examined the distribution of CYP26A1 protein by double immunostaining of CYP26A1 with the neuronal marker NeuN, the astrocytic marker GFAP, and the microglial marker IBA-1. The results showed that CYP26A1-immunoreactive staining was mainly colocalized with NeuN, partially with GFAP, and a few with IBA-1 (Fig. 2I–L), indicating the distribution of CYP26A1 in spinal neurons and glial cells.

### The Concentration of at-RA Is Decreased in the Spinal Cord After SNL

**Fig. 3:**
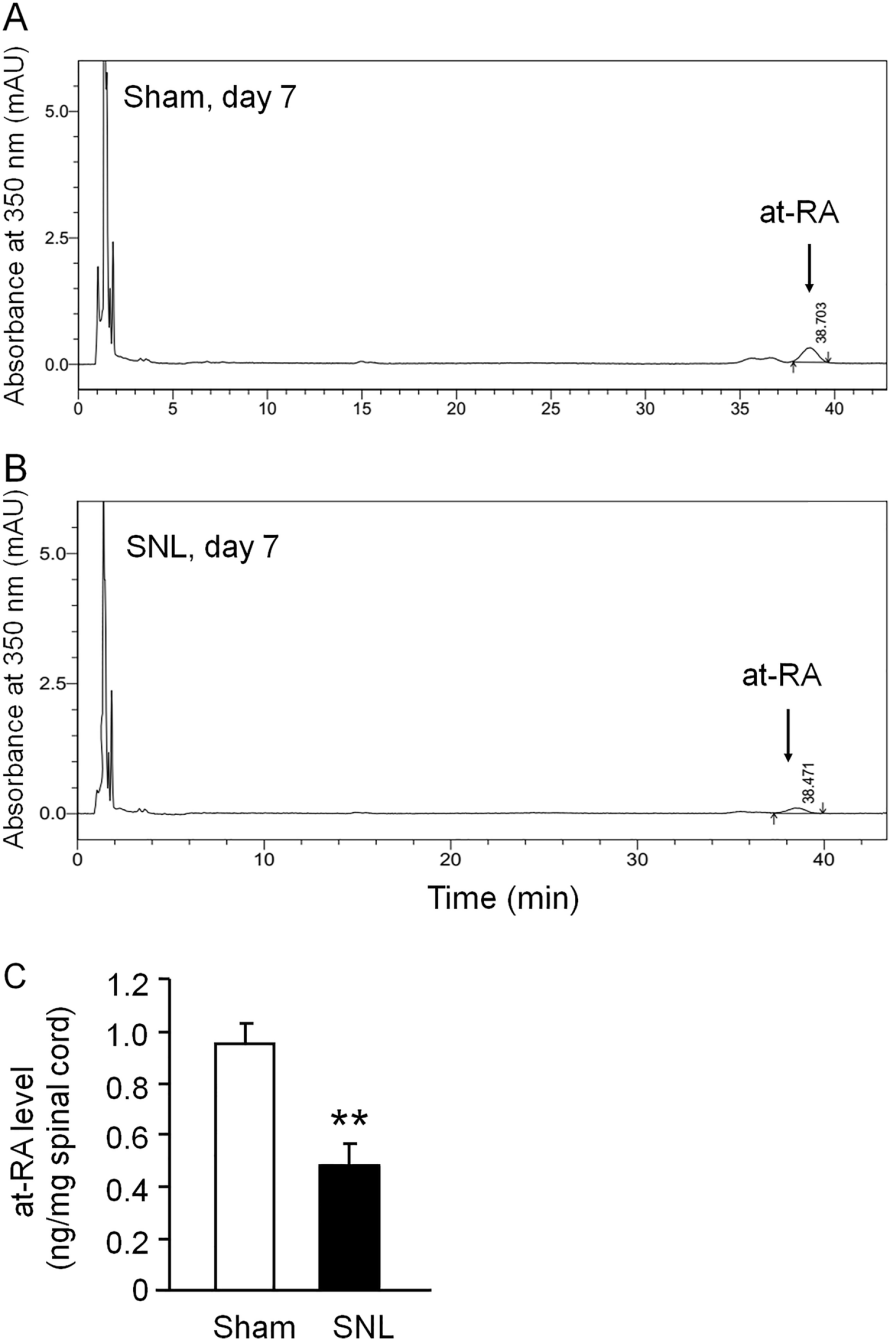
The concentration of at-RA is decreased in the spinal cord after SNL. **A**, **B** HPLC chromatograms of at-RA in the spinal cord in Sham- (**A**) and SNL-operated (**B**) mice. **C** Spinal at-RA concentration (ng/mg) is lower in SNL mice than in Sham-operated mice. **P <0.01, Student’s *t*-test, *n* = 3 group.

As at-RA is the preferred substrate of CYP26A1 [40], we checked the concentration of at-RA in the spinal cord after SNL. The ipsilateral spinal cords were harvested 7 days after SNL or sham operation. HPLC analysis showed that the major peak of at-RA absorbance at 350 nm was detected at 38 min (Fig. 3A, B). The concentration of the at-RA was calculated according to the at-RA standard curve. The level of spinal at-RA was significantly decreased on day 7 in SNL mice compared with sham-operated mice (*P* <0.01, Student’s *t*-test, Fig. 3C). These results suggest that the increased CYP26A1 may cause the decrease of at-RA after SNL.

### Intrathecal Injection of *Cyp26a1* siRNA Attenuates SNL-Induced Neuropathic Pain

**Fig. 4:**
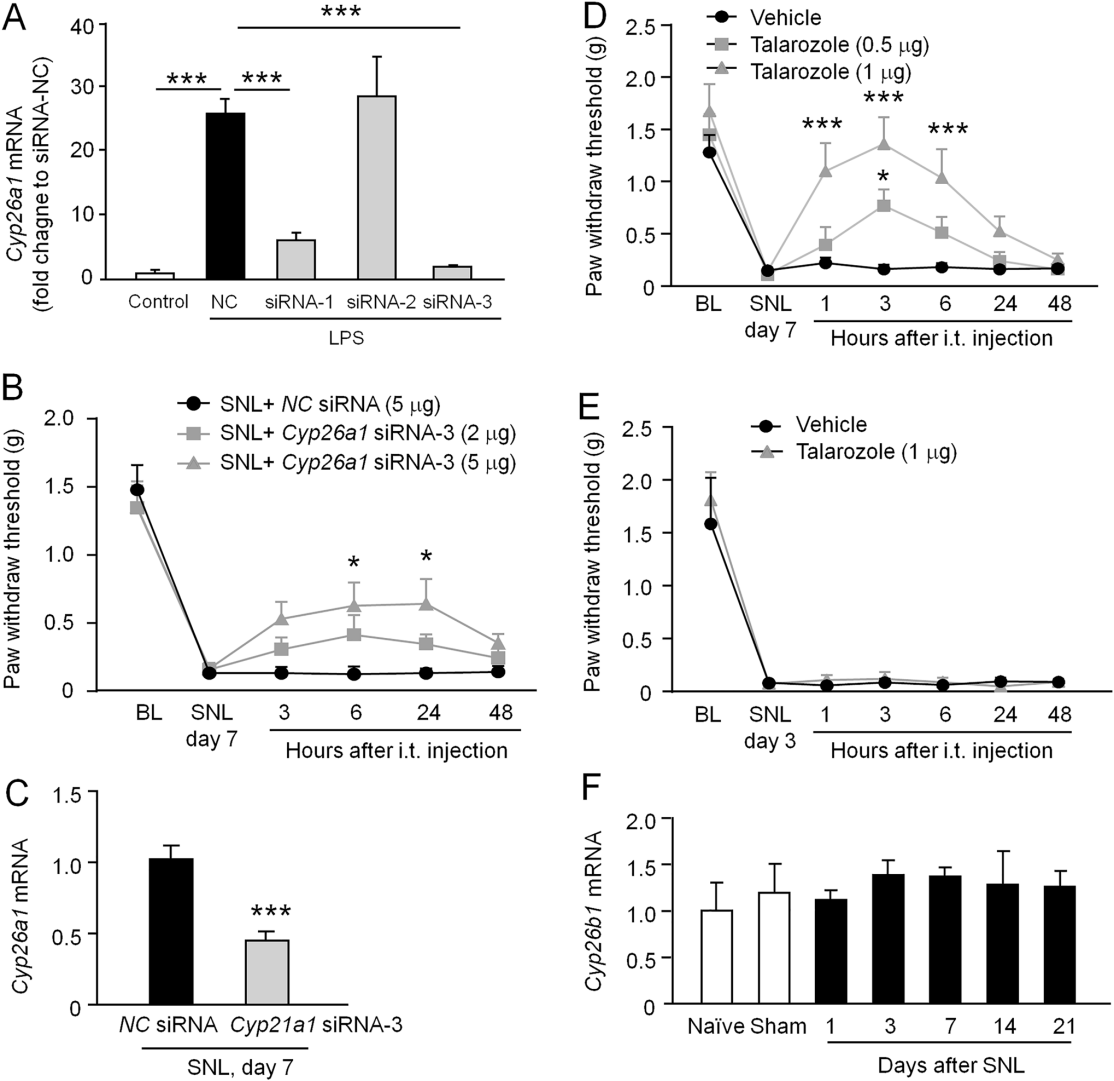
Inhibition of CYP26A1 expression or CYP26A1-mediated at-RA catabolism attenuates SNL-induced neuropathic pain. **A** qPCR shows that LPS-induced upregulation of *Cyp26a1* mRNA is reduced by pretreatment with siRNA-1 or siRNA-3 in cultured astrocytes. ***P <0.001, one-way ANOVA followed by Bonferroni’s test. *n* = 4/group. **B** Intrathecal injection of siRNA-3 (5 μg) attenuates SNL-induced mechanical allodynia. *P <0.05 *vs* SNL + NC-siRNA, two-way repeated measures ANOVA followed by Bonferroni’s test. *n* = 6–8 mice/group. **C** qPCR showing the decreased *Cyp26a1* mRNA in the spinal cord after siRNA-3 (5 μg) treatment. ***P <0.001, Student’s *t*-test. *n* = 6 mice/group. **D** Injection of talarozole 7 days after SNL dose-dependently alleviates SNL-induced mechanical allodynia. *P <0.05, ***P <0.001, talarozole *vs* vehicle, two-way repeated measures ANOVA followed by Bonferroni’s test. *n* = 8–10 mice/group. **E** Injection of talarozole (1 μg) at 3 days after SNL does not affect SNL-induced mechanical allodynia. Two-way repeated measures ANOVA followed by Bonferroni’s test. *n* = 5–6 mice/group. **F** qPCR showing the time course of *Cyp26b1* mRNA expression in the spinal cord after SNL. One-way ANOVA followed by Bonferroni’s test. *n* = 5–6 mice/group.

We then examined the role of CYP26A1 in SNL-induced neuropathic pain. First, we tested the knockdown effect of three *Cyp26a1* siRNAs using cultured primary astrocytes. *Cyp26a1* mRNA was increased after stimulation by LPS. Pretreatment with *Cyp26a1* siRNA-1 or siRNA-3 inhibited the LPS-induced upregulation of *Cyp26a1* mRNA by 76.6% ± 5.3% and 92.4% ± 0.6% compared with the NC siRNA, respectively (*F*_(4, 15)_ = 34.01, *P* <0.001, one-way ANOVA followed by Bonferroni’s test, Fig. 4A). In contrast, *Cyp26a1* siRNA-2 had no interference effect. Based on these data, we chose the *Cyp26a1* siRNA-3 for further *in vivo* study. Meanwhile, *Cyp26a1* siRNA-3 and NC siRNA were modified with 2'-methoxy (2'-OMe) and cholesterol to increase their water solubility and stability.

Mechanical allodynia is a cardinal feature of neuropathic pain. We then checked the mechanical allodynia after intrathecal injection of siRNAs (2 μg or 5 μg) at SNL Day 7 when *Cyp26a1* mRNA peaked. The results showed that the higher dose of *Cyp26a1* siRNA-3 (5 µg) partly attenuated the SNL-induced mechanical allodynia from 6 h to 24 h after injection, but the low dose (2 µg) did not have a significant antiallodynic effect (*F*_(2, 18)_ = 3.875, *P* <0.05, two-way repeated measures ANOVA, Fig. 4B). To confirm the knockdown effect of *Cyp26a1* siRNA-3 *in vivo*, we checked the *Cyp26a1* mRNA expression 24 h after injection using other animals. The *Cyp26a1* mRNA expression was reduced by 57.0 ± 8.2% following the *Cyp26a1* siRNA-3 treatment compared to the NC siRNA (*P* <0.001, Student’s *t*-test, Fig. 4C). These results suggest that CYP26A1 is involved in the maintenance of neuropathic pain.

### Intrathecal Injection of the at-RA Metabolism-blocking Agent Talarozole Alleviates SNL-Induced Neuropathic Pain

To further determine whether CYP26A1 contributes to neuropathic pain *via* regulating the at-RA level, we intrathecally injected talarozole (R115866), a potent and selective at-RA metabolism blocking agent, which inhibits both CYP26A1 and CYP26B1. As shown in Fig. 4D, talarozole dose-dependently alleviated the mechanical allodynia at 7 days after SNL. The anti-allodynic effect became evident from 1 h to 6 h after injection of the higher dose of talarozole (*F*_(2, 25)_ = 15.37, *P* <0.0001, two-way repeated measures ANOVA, Fig. 4D). A lower dose of talarozole (0.5 µg) attenuated the mechanical allodynia at 3 h after injection. However, SNL-induced mechanical allodynia was not attenuated by this treatment 3 days after SNL (*F*_(1, 9)_ = 0.2517, *P* >0.05, two-way repeated measures ANOVA, Fig. 4E). Since talarozole also inhibits CYP26B1, we checked the time course of *Cyp26b1* mRNA expression in the spinal cord after SNL. There was no significant difference between naïve, sham-operated, and SNL mice, suggesting that CYP26B1 may not be involved in neuropathic pain (*F*_(6, 33)_ = 0.3594, *P* >0.05, one-way ANOVA. Fig. 4F). These data collectively indicate that CYP26A1 is involved in the maintenance but not the development of neuropathic pain.

### Talarozole Inhibits SNL-Induced Neuronal and Glial Activation

**Fig. 5:**
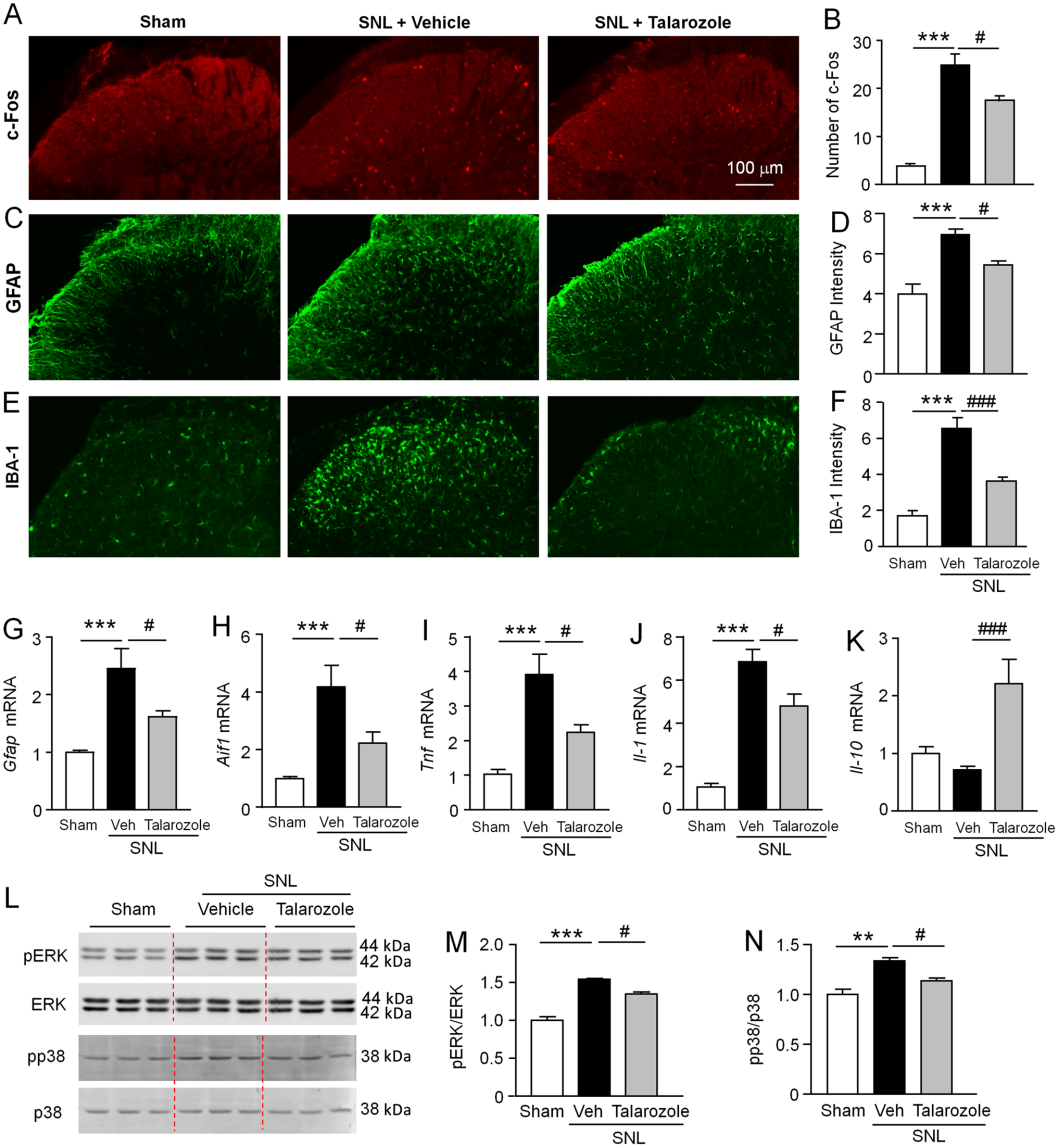
Talarozole reduces SNL-induced neuronal and glial activation. **A**–**F** Immunofluorescence staining and intensity histogram of c-Fos (**A, B**), GFAP (**C, D**), and IBA-1 (**E, F**) in the ipsilateral spinal dorsal horn in Sham, SNL+ vehicle, and SNL + talarozole-groups. ***P <0.001, SNL + vehicle *vs* Sham; ^**#**^*P* <0.05, ^**###**^*P* <0.001, SNL + talarozole *vs* SNL +Vehicle, one-way ANOVA followed by Bonferroni’s test. *n* = 4–5 mice/group. Scale bar, 100 μm (**A**, **C**, and **E**). **G**–**K** qPCR show that injection of talarozole (1 μg) 7 days after SNL decreases the mRNA levels of *Gfap* (**G**), *Aif1* (**H**), *Tnf* (**I**), and *Il-1* (**J**) in the spinal cord. Talarozole increases *Il-10* mRNA compared to vehicle treatment (**K**). ***P <0.001, SNL + Vehicle *vs* Sham; ^**#**^*P* <0.05, ^**###**^*P* <0.001, SNL + Talarozole *vs* SNL + Vehicle, one-way ANOVA followed by Bonferroni’s test. *n* = 6–9 mice/group. **L**–**N** Western blots for pERK and pp38 show that talarozole reduces SNL-induced pERK and pp38 upregulation in the spinal cord. **P <0.01, ***P <0.001, SNL + Vehicle *vs* Sham; ^**#**^*P* <0.05, SNL + Talarozole *vs* SNL + Vehicle, one-way ANOVA followed by Bonferroni’s test. *n* = 3 mice/group.

To explore the mechanism of CYP26A1 in neuropathic pain, we measured the expression of the neuronal activation marker c-Fos, the astrocytic marker GFAP, and the microglial marker IBA-1 in the spinal cord. Immunostaining showed that SNL-induced c-Fos expression was decreased after talarozole treatment (1 µg) on SNL day 7 (*F*_(2, 9)_ = 32.66, *P* <0.001, one-way ANOVA followed by Bonferroni’s test, Fig. 5A, B). In addition, talarozole also significantly reduced the SNL-induced increase of GFAP-IR and IBA-1-IR, compared to vehicle (GFAP-IR, *F*_(2, 12)_ = 18.82, *P* <0.001; IBA-1-IR, *F*_(2, 12)_ = 37.71, *P* <0.001, one-way ANOVA followed by Bonferroni’s test, Fig. 5C–F).

We further assessed the expression of GFAP and IBA-1 by qPCR. The results showed that the mRNA levels of *Gfap* and *Aif1* (the gene of IBA-1) were increased after SNL. Talarozole treatment decreased *Gfap* mRNA (*F*_(2, 23)_ = 11.7, *P* <0.001, one-way ANOVA followed by Bonferroni’s test, Fig. 5G) and *Aif1* mRNA (*F*_(2, 23)_ = 11.07, *P* <0.001, one-way ANOVA followed by Bonferroni’s test, Fig. 5H). As at-RA can regulate inflammatory cytokine expression *in vitro* [29, 32, 33], we examined the levels of the inflammatory cytokines *Tnf* and *Il-1* and the anti-inflammatory cytokine *Il-10* using the same samples. This showed that talarozole reduced the increase of *Tnf* and *Il-1* by SNL (*Tnf*, *F*_(2, 22)_ = 14.56, *P* <0.001; *Il-1*, *F*_(2, 21)_ = 48.1, *P* <0.001, one-way ANOVA followed by Bonferroni’s test, Fig. 5I, J). Meanwhile, SNL did not significantly change the *Il-10* level, whereas talarozole treatment increased it (*F*_(2, 19)_ = 10.97, *P* <0.001, one-way ANOVA followed by Bonferroni’s test, Fig. 5K). These data indicate that talarozole reduces neuroinflammation in the spinal cord.

ERK and p38 are important intracellular kinases that are involved in neuropathic pain [41]. ERK is activated in spinal neurons 1 day after SNL and in astrocytes and microglia 10 days after SNL [42], whereas p38 is exclusively activated in spinal microglia in mice [43]. We further checked the level of pERK and pp38 with or without talarozole treatment. Western blots showed that SNL increased pERK and pp38 levels, which were decreased by the treatment with talarozole (pERK, *F*_(2, 6)_ = 68.46, *P* <0.001; pp38, *F*_(2, 6)_ = 19.62, *P* <0.01, one-way ANOVA followed by Bonferroni’s test, Fig. 5L–N). These data support the conclusion that CYP26A1 contributes to SNL-induced glial activation and neuroinflammation in the spinal cord.

### RA Receptor Expression in the Spinal Cord After SNL

**Fig. 6:**
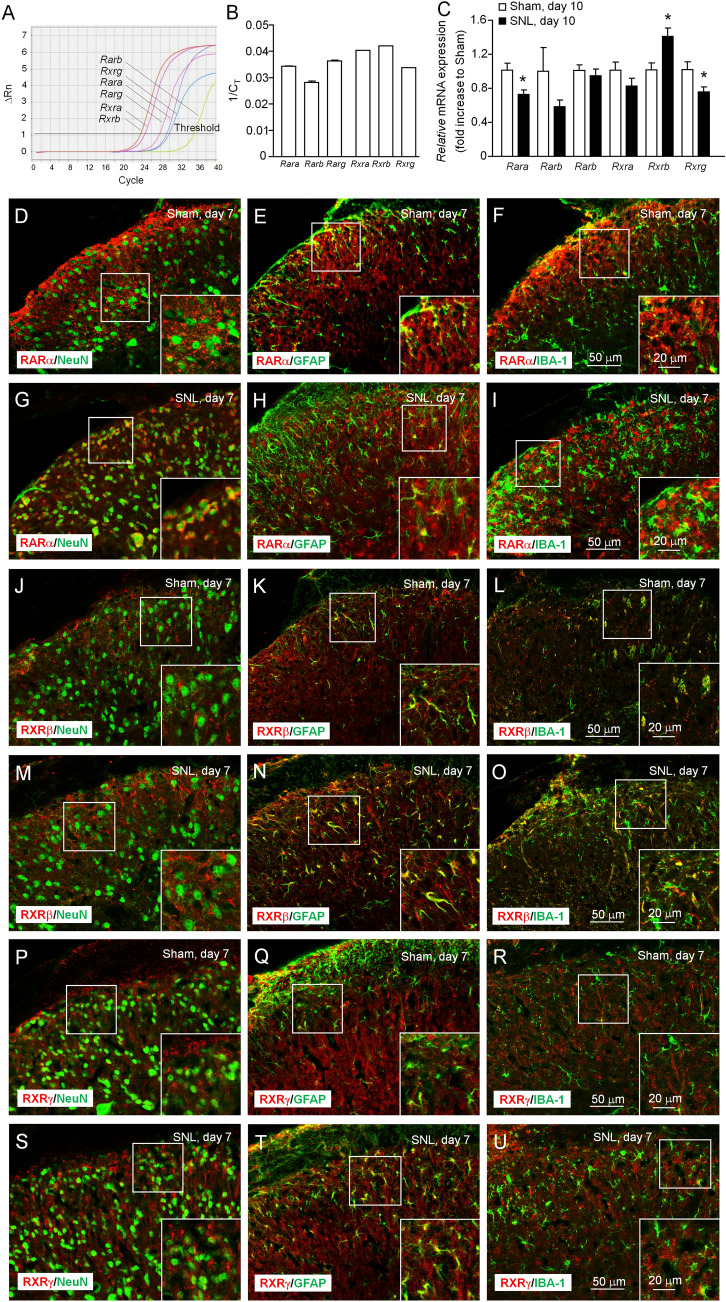
RA receptor expression in the spinal cord. **A** Real-time quantitative PCR (qPCR) show the expression of *Rara*, *Rarb*, *Rarg*, *Rxra*, *Rxab*, and *Rxrg* mRNA in the spinal cord of naïve mice. **B** Histogram showing the level of the 6 genes. *n* = 6 mice/group. **C** qPCR showing that *Rara* and *Rxrg* mRNA are decreased, and *Rxrb* mRNA is increased 10 days after SNL. *P <0.05, Student’s *t*-test. *n* = 4–6 mice/group. **D-F** Double staining of RARα with NeuN (**D**), GFAP (**E**), and IBA-1 (**F**) in the dorsal horn of sham-operated mice. **G**–**I** Double staining of RARα with NeuN (**G**), GFAP (**H**), and IBA-1 (**I**) in the dorsal horn of SNL-operated mice. **J**–**L** Double staining of RXRβ with NeuN (**J**), GFAP (**K**), and IBA-1 (**L**) in the dorsal horn of sham-operated mice. **M-O** Double staining of RXRβ with NeuN (**M**), GFAP (**N**), and IBA-1 (**O**) in the dorsal horn of SNL-operated mice. **P**–**R** Double staining of RXRγ with NeuN (**P**), GFAP (**Q**), and IBA-1 (**R**) in the dorsal horn of sham-operated mice. **S**–**U** Double staining of RXRγ with NeuN (**S**), GFAP (**T**), and IBA-1 (**U**) in the dorsal horn of SNL-operated mice. Scale bars, 50 μm (**D–U**). Scale bar for the inset boxes (**D–U**), 20 μm.

RA exerts its effects by binding to retinoic acid receptors (RARs, including RARα, RARβ, and RARγ) which heterodimerize with retinoid X receptors (RXRs, including RXRα, RXRβ, and RXRγ) [44]. The qPCR showed that *Rara*, *Rarg*, *Rxra*, *Rxrb*, and *Rxrg* were constitutively expressed in the spinal cord, whereas *Rarb* had a very low level (Fig. 6A, B). Ten days after SNL, the *Rara* and *Rxrg* mRNA levels were significantly decreased, *Raxb* mRNA was increased, and the other RA receptors were not significantly changed (*P* <0.05, Student’s *t*-test, Fig. 6C), indicating that SNL mildly regulates the mRNA levels of three RA receptors.

We then examined the distribution of RARα, RXRβ, and RXRγ in the spinal cord. Double staining showed that RARα was mainly colocalized with GFAP and IBA-1 in sham-operated mice (Fig. 6D–F), but colocalized with NeuN and GFAP after SNL (Fig. 6G–I). RXRβ was colocalized with GFAP and IBA-1 in both sham- and SNL-operated mice (Fig. 6J–O). In contrast, RXRγ was mainly colocalized with GFAP, and a few cells with IBA-1 in sham- and SNL-operated mice (Fig. 6P–U). These data suggest that RARα, RXRβ, and RXRγ are differently distributed in neurons, astrocytes, and microglia in the spinal cord.

### At-RA Promotes the Expression of IL-10 by which Alleviates Neuropathic Pain

**Fig. 7:**
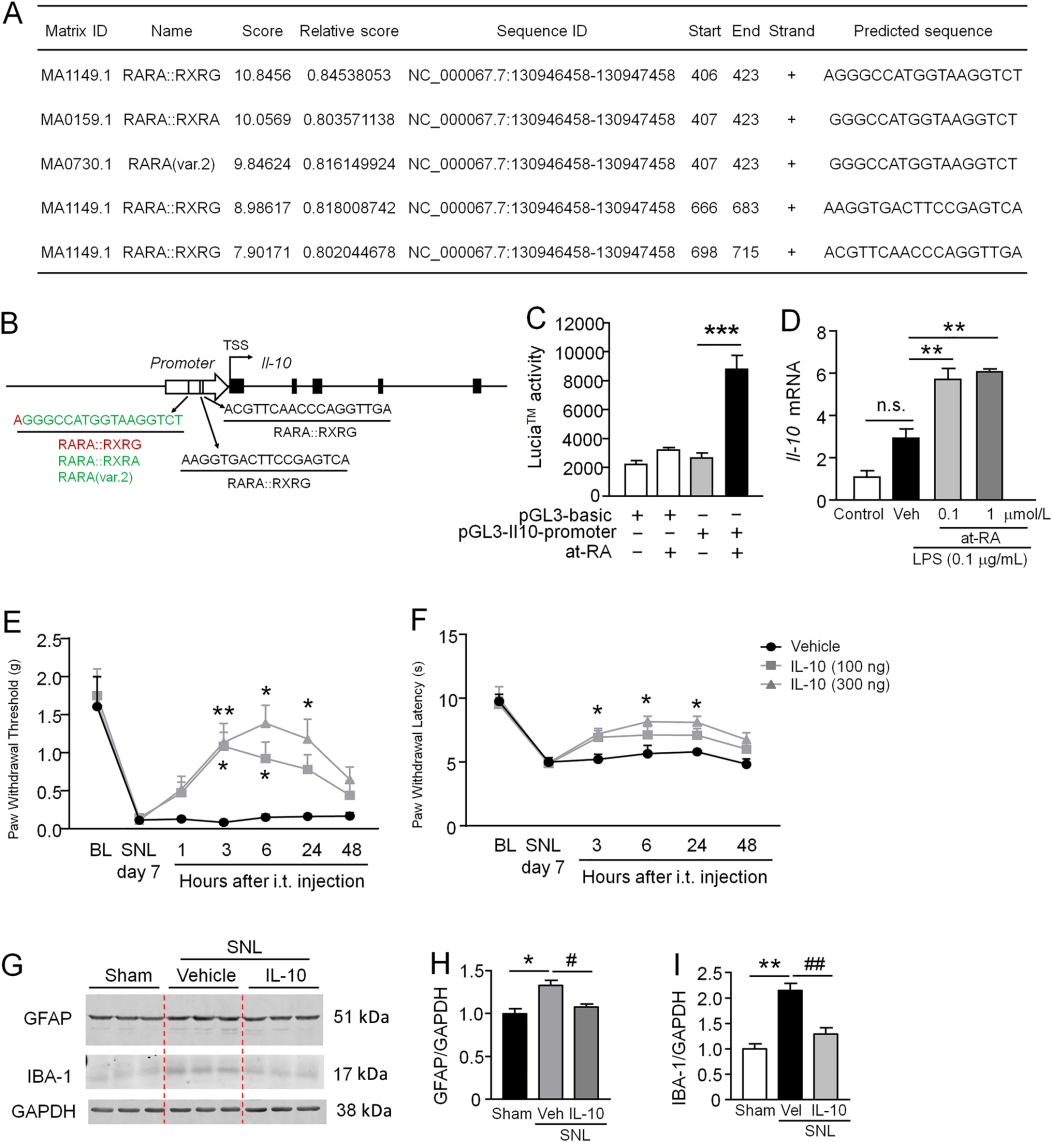
RA receptors regulate the expression of IL-10, and the latter attenuates SNL-induced neuropathic pain and glial activation. **A** Analysis of at-RA receptor (RARs and RXRs) binding sites in the mouse *Il-10* promoter sequence from the transcription start site (TSS) upstream −1000 to −1. **B** Schematic illustrating at-RA receptor (RARs and RXRs) binding sites in the *Il-10* promoter sequence. **C** Luciferase reporter assay shows that at-RA treatment upregulates the transcription activity of the *Il-10* gene promoter. The values are normalized to *Renilla* luciferase activity. ***P <0.001, basic vector + at-RA *vs Il-10* promoter vector + at-RA, one-way ANOVA followed by Bonferroni’s test. *n* = 4/group. **D** qPCR shows that treatment with at-RA (0.1 μmol/L) for 12 h increases the LPS-induced expression of *Il-10* mRNA at 6 h after stimulation. **P <0.01, one-way ANOVA followed by Bonferroni’s test. *n* = 3/group. n.s. no significant difference. **E, F** Intrathecal injection of IL-10 attenuates SNL-induced mechanical allodynia (**E**) and heat hyperalgesia (**F**). *P <0.05, **P <0.01 two-way repeated measures ANOVA followed by Bonferroni’s test. *n* = 5–7 mice/group. **G**–**I** IL-10 reduces SNL-induced GFAP and IBA-1 upregulation in the spinal cord. *P <0.05, **P <0.01, SNL + Vehicle *vs* Sham; ^**#**^*P* <0.05, ^**##**^*P* <0.01, SNL + talarozole *vs* SNL + Vehicle, one-way ANOVA followed by Bonferroni’s test. *n* = 3 mice/group.

As RA receptors may act as ligand-activated transcription factors to regulate gene expression [45, 46], and talarozole reduced TNF-α and IL-1β and increased IL-10 in the spinal cord after SNL (Fig. 4I–K), we asked if RA receptors directly regulate the expression of *Tnf*, *Il-1*, and *Il-10*. We analyzed the sequence from –1000 to –1 of the promoters of these three genes. There is no binding site for RA receptors on the *Tnf* promoter, 2 binding sites on the *Il-1* promoter, and 5 binding sites on the *Il-10* promoter based on JASPAR CORE in Vertebrata with a defined 80% profile score threshold (http://jaspar.genereg. net/) (Fig. 7A). Furthermore, the *Il–10* promoter has 3 binding sites for RARA::RXRG, 1 binding site for RARA::RXRA, and 1 binding site for RARA(var.2) (Fig.7B). To determine the effect of at-RA on *Il-10* expression, we applied a luciferase activity assay *in vitro*. pGL3-basic vector or pGL3-*Il10*-promoter-Lucia vector was transfected into BV2 microglia cells for 24 h, followed by incubation of at-RA (0.1 μmol/L) for 12 h. As shown in Fig. 7C, in BV2 cells transfected with the pGL3-Il10-promoter-Lucia vector, treatment with at-RA significantly increased the promoter luciferase activity compared with the pGL3-basic vector (*F*_(3, 12)_ = 32.99, *P* <0.001, one-way ANOVA followed by Bonferroni’s test). In addition, the effects of different concentrations of at-RA (0.1 and 1 μmol/L) on *Il-10* mRNA expression were also determined in BV2 cells by qPCR. The results showed that pretreatment with at-RA for 12 h increased the LPS-induced expression of *Il-10* mRNA (*F*_(3, 8)_ = 38.29, *P* <0.001, one-way ANOVA followed by Bonferroni’s test. Fig. 7D).

Finally, we intrathecally injected IL-10 at SNL day 7 and tested pain behaviors. The results showed that IL-10 dose-dependently attenuated the SNL-induced mechanical allodynia (*F*_(2, 14)_ = 4.499, *P* <0.05, two-way RM ANOVA followed by Bonferroni’s test, Fig. 7E) and heat hyperalgesia (*F*_(2, 14)_ = 7.842, *P* <0.01, two-way RM ANOVA followed by Bonferroni’s test, Fig. 7F). Furthermore, Western blots showed that intrathecal injection of IL-10 (100 ng) reduced the SNL-induced upregulation of GFAP (*F*_(2, 6)_ = 10.85, *P* <0.05, one-way ANOVA followed by Bonferroni’s test, Fig. 7G, H) and IBA-1 (*F*_(2, 6)_ = 23.88, *P* <0.01, one-way ANOVA followed by Bonferroni’s test. Fig. 7G, I). These data suggest that RA receptors regulate IL-10 expression and contribute to the maintenance of neuropathic pain.

## Discussion

The present study provides novel evidence that CYP26A1, one of the CYP450s functions in the maintenance of neuropathic pain. CYP26A1 was upregulated in neurons and glial cells of the spinal cord after SNL. In addition, the increased CYP26A1 was associated with the decreased at-RA level in the spinal cord. Inhibition of CYP26A1 expression by siRNA or inhibition of CYP26A1-mediated at-RA metabolization by talarozole attenuated the SNL-induced mechanical allodynia. Talarozole also decreased the activation of astrocytes and microglia, reduced the expression of *Tnf* and *Il-1*, and promoted the expression of *Il-10* mRNA in the spinal cord. Meanwhile, RA receptors were expressed in spinal neurons and glial cells and directly regulated *Il-10* mRNA expression. Finally, intrathecal injection of IL-10 attenuated SNL-induced neuropathic pain and glial activation. Taken together, our results indicate that CYP26A1 contributes to neuropathic pain by reducing the at-RA level and IL-10 production, thus maintaining glial activation in the spinal cord.

### CYP26A1 Contributes to SNL-Induced Mechanical Allodynia

A previous study showed that CYP2J6 is increased in the dorsal root ganglia of paclitaxel-treated mice and contributes to chemotherapy-induced mechanical hypersensitivity [47]. Whether other CYP450 family members are involved in neuropathic pain is less studied. Our array data showed that CYP26A1, CYP4B1, and CYP1B1 were upregulated more than 7-fold in the spinal cord after SNL. CYP4B1 and CYP1B1 are both expressed in the liver and extrahepatic tissues to carry out the metabolism of numerous xenobiotics [48, 49]. CYP4B1 is expressed in various cancers and plays a role in cancer development *via* the activation of procarcinogens and neovascularization [49]. CYP1B1 is involved in obesity, hypertension, adipogenesis, and atherosclerosis *via* regulating endogenous metabolic pathways including the metabolism of steroid hormones, fatty acids, and vitamins [48]. In this study, the increased CYP26A1, CYP1B1, and CYP4B1 suggest a potential role of these CYP450 family members in neuropathic pain. Here, we focused on CYP26A1 and confirmed a marked increase of CYP26A1 in the spinal cord 7 days and 14 days after SNL. Consistent with this, our HPLC analysis displayed a decreased concentration of spinal at-RA in this phase, indicating a role of CYP26A1 in maintaining the at-RA level in the spinal cord.

Previous studies have shown that at-RA attenuates neuroinflammation in rat brains [50] and prevents amyloidogenesis and memory impairment in aged rats [51]. However, the role of RA in the treatment of chronic pain remains controversial. Romero-Sandoval *et al.* showed that the oral administration of at-RA enhances nociceptive withdrawal reflexes in rats with soft-tissue inflammation [52]. In contrast, Hamed *et al.* reported that intraperitoneal administration of RA attenuates the neuropathic pain induced by chronic constriction injury of the sciatic nerve in rats [53]. The difference may be due to the different doses or different means of administration. As systemic administration of RA has both peripheral and central effects, we intrathecally injected *Cyp26a1* siRNA and talarozole to test the central effect. Specific knockdown of *Cyp26a1* in the spinal cord effectively attenuated SNL-induced mechanical allodynia. Intrathecal injection of talarozole at SNL day 7 but not day 3 alleviated the mechanical allodynia, indicating the involvement of CYP26A1 in the maintenance of neuropathic pain. It has been reported that oral administration of R115866 (talarozole) induces marked increases in RA levels in the plasma and skin [54]. Talarozole was developed to treat psoriasis and acne [55, 56]. The present study suggests that talarozole represents a promising strategy for the treatment of established neuropathic pain.

### CYP26A1 is Involved in SNL-Induced Neuronal and Glial Activation

RA binds to RARs and RXRs which then act as ligand-activated transcription factors to regulate gene expression [45, 46]. It has been reported that RARα, RXRα, RXRβ, and RXRγ are localized in the neurons, and RXRα and RXRβ are expressed in the astrocytes of naïve rats [57]. Here, SNL decreased the *Rara* and *Rxrg* mRNA levels and increased the *Rxrb* mRNA levels. Moreover, RARα was mainly expressed in the astrocytes of sham-operated mice, but was expressed in neurons and astrocytes after SNL. In contrast, RXRβ was mildly expressed in astrocytes and microglia in sham-operated mice and increased in these cell types after SNL. RXRγ was dominantly expressed in spinal astrocytes and had low expression in the microglia under both normal and neuropathic pain conditions. Our results also showed that CYP26A1 was predominantly expressed in spinal neurons and astrocytes, thus the increased CYP26A1 after SNL may cause the decrease of at-RA in these cells. As RA can leave the cell through the plasma membrane and bind to cytosolic binding proteins, enters the nucleus, and function in a paracrine and an autocrine manner in the adult brain [58–60], RA may act *via* different receptors in neurons, astrocytes, and microglia of the spinal cord. Consistent with this, immunostaining showed that inhibition of CYP26A1 degradation of at-RA by talarozole substantially reduced the SNL-induced upregulation of c-Fos, GFAP, and IBA-1. Western blots showed that the pERK and pp38 upregulated by SNL were also decreased by talarozole. As pERK is expressed in spinal astrocytes and microglia, and pp38 is expressed in spinal microglia 10 days after SNL [42, 43], the reduced pERK and pp38 support the reduced activation of astrocytes and microglia. It has been well-demonstrated that microglia, astrocytes, and neurons have close cross-talk which plays an important role in the pathogenesis of neuropathic pain [61, 62]. For example, pineal neurons release CX3CL1 to activate microglia *via* CX3CR1 after SNL [63, 64]. Optogenetic activation of astrocytes elicits microglial activation [65], and inhibition of SNL-induced astrocytic activation by deletion of *Cxcr5* reduces microglial activation [38]. Therefore, the reduced RA by CYP26A1 contributes to increased spinal neuroinflammation and neuropathic pain.

### At-RA Increases IL-10 Expression by Which Glial Activation is Inhibited and Neuropathic Pain is Attenuated

Previous studies have shown that at-RA inhibits the neurotoxic effect on microglia by suppressing the expression levels of TNF-α and iNOS induced by LPS [33]. The addition of at-RA potentiates the LPS-induced IL-10 mRNA expression and the number of IL-10-secreting cells from the THP-1 monocyte/macrophage cell line and cord blood mononuclear cells [32], but the regulatory mechanism of at-RA on cytokine expression has not been studied. Here the bioinformatics analysis showed that RARA:RXRG, RARA:RXRA, and RARA(var2) directly bind on the *Il-10* promoter. The luciferase activity assay indicated the increased expression of *Il-10* by at-RA. *In vivo,* data showed that intrathecal talarozole increased the *Il-10* level in the spinal cord. However, which heterodimer or homodimer plays a major role in IL-10 expression and whether RA receptors regulate Il-1β expression still needs further investigation.

IL-10 has been well demonstrated to have anti-inflammatory and anti-nociceptive effects in animal models of neuropathic pain, peripheral diabetic pain, bone cancer pain, and inflammatory pain [66–70]. In agreement with these reports, intrathecal IL-10 attenuated SNL-induced pain hypersensitivity and reduced SNL-induced activation of astrocytes and microglia. It has been reported that IL-10 reduces TNF-α and IL-1β production and counter-regulates the actions of TNF-α and IL-1β by increasing TNF decoy receptors and IL-1 receptor antagonists [71]. TNF-α and IL-1β are important in mediating nerve injury-induced glial activation and enhancing synaptic transmission [72, 73]. IL-10 suppresses the enhanced frequency and amplitude of the miniature excitatory postsynaptic currents in the spinal dorsal horn neurons of lamina II in neuropathic rats [70]. Taken together, the increased CYP26A1 may reduce IL-10 production and contributes to spinal neuroinflammation and neuropathic pain.

In summary, this study revealed that a CYP450 family member, CYP26A1, is increased in spinal neurons and astrocytes after SNL. Meanwhile, at-RA, the preferred substrate of CYP26A, was decreased. The RA receptors such as RARα, RXRβ, and RXRγ homodimers or heterodimers directly bind the *Il-10* promoter and regulate IL-10 production. Inhibition of CYP26A1 by siRNA or an inhibitor increased IL-10 production, reduced TNF-α and IL-1β production, and decreased SNL-induced glial activation and neuropathic pain. Thus, CYP26A1 may be a potential target for the treatment of neuropathic pain.

## References

[1] Nelson DR, Koymans L, Kamataki T, Stegeman JJ, Feyereisen R, Waxman DJ (1996). P450 superfamily: Update on new sequences, gene mapping, accession numbers and nomenclature. Pharmacogenetics.

[2] Guengerich FP (1997). Comparisons of catalytic selectivity of cytochrome P450 subfamily enzymes from different species. Chem Biol Interact.

[3] Nebert DW, Gonzalez FJ (1987). P450 genes: Structure, evolution, and regulation. Annu Rev Biochem.

[4] Lu Y, Cederbaum AI (2018). Cytochrome P450s and alcoholic liver disease. Curr Pharm Des.

[5] Rasheed MSU, Mishra AK, Singh MP (2017). Cytochrome P450 2D6 and parkinson’s disease: Polymorphism, metabolic role, risk and protection. Neurochem Res.

[6] Song BJ, Abdelmegeed MA, Cho YE, Akbar M, Rhim JS, Song MK (2019). Contributing roles of CYP2E1 and other cytochrome P450 isoforms in alcohol-related tissue injury and carcinogenesis. Adv Exp Med Biol.

[7] Gilroy DW, Edin ML, De Maeyer RP, Bystrom J, Newson J, Lih FB (2016). CYP450-derived oxylipins mediate inflammatory resolution. Proc Natl Acad Sci U S A.

[8] Lutz JD, Dixit V, Yeung CK, Dickmann LJ, Zelter A, Thatcher JE (2009). Expression and functional characterization of cytochrome P450 26A1, a retinoic acid hydroxylase. Biochem Pharmacol.

[9] Ross AC, Zolfaghari R (2011). Cytochrome P450s in the regulation of cellular retinoic acid metabolism. Annu Rev Nutr.

[10] Duester G (2008). Retinoic acid synthesis and signaling during early organogenesis. Cell.

[11] Gudas LJ, Wagner JA (2011). Retinoids regulate stem cell differentiation. J Cell Physiol.

[12] Maden M (2007). Retinoic acid in the development, regeneration and maintenance of the nervous system. Nat Rev Neurosci.

[13] Gudas LJ (2012). Emerging roles for retinoids in regeneration and differentiation in normal and disease states. Biochim Biophys Acta.

[14] Abu-Abed S, Dollé P, Metzger D, Beckett B, Chambon P, Petkovich M (2001). The retinoic acid-metabolizing enzyme, CYP26A1, is essential for normal hindbrain patterning, vertebral identity, and development of posterior structures. Genes Dev.

[15] Bowles J, Knight D, Smith C, Wilhelm D, Richman J, Mamiya S (2006). Retinoid signaling determines germ cell fate in mice. Science.

[16] Koubova J, Menke DB, Zhou Q, Capel B, Griswold MD, Page DC (2006). Retinoic acid regulates sex-specific timing of meiotic initiation in mice. Proc Natl Acad Sci USA.

[17] Sakai Y, Meno C, Fujii H, Nishino J, Shiratori H, Saijoh Y (2001). The retinoic acid-inactivating enzyme CYP26 is essential for establishing an uneven distribution of retinoic acid along the anterio-posterior axis within the mouse embryo. Genes Dev.

[18] Ricard MJ, Gudas LJ (2013). Cytochrome p450 cyp26a1 alters spinal motor neuron subtype identity in differentiating embryonic stem cells. J Biol Chem.

[19] Larsen R, Proue A, Scott EP, Christiansen M, Nakagawa Y (2019). The thalamus regulates retinoic acid signaling and development of parvalbumin interneurons in postnatal mouse prefrontal cortex. eNeuro.

[20] Wang Y, Zolfaghari R, Ross AC (2002). Cloning of rat cytochrome P450RAI (CYP26) cDNA and regulation of its gene expression by all-*trans*-retinoic acid *in vivo*. Arch Biochem Biophys.

[21] Xi J, Yang Z (2008). Expression of RALDHs (ALDH1As) and CYP26s in human tissues and during the neural differentiation of P19 embryonal carcinoma stem cell. Gene Expr Patterns.

[22] White JA, Ramshaw H, Taimi M, Stangle W, Zhang A, Everingham S (2000). Identification of the human cytochrome P450, P450RAI-2, which is predominantly expressed in the adult cerebellum and is responsible for all-trans-retinoic acid metabolism. Proc Natl Acad Sci USA.

[23] Reijntjes S, Gale E, Maden M (2004). Generating gradients of retinoic acid in the chick embryo: Cyp26C1 expression and a comparative analysis of the Cyp26 enzymes. Dev Dyn.

[24] Tahayato A, Dolle P, Petkovich M (2003). Cyp26C1 encodes a novel retinoic acid-metabolizing enzyme expressed in the hindbrain, inner ear, first branchial arch and tooth buds during murine development. Gene Expr Patterns.

[25] Zhang Y, Crofton EJ, Smith TES, Koshy S, Li D, Green TA (2019). Manipulation of retinoic acid signaling in the nucleus accumbens shell alters rat emotional behavior. Behav Brain Res.

[26] Donnelly CR, Andriessen AS, Chen G, Wang K, Jiang C, Maixner W (2020). Central nervous system targets: Glial cell mechanisms in chronic pain. Neurotherapeutics.

[27] Lu HJ, Gao YJ (2023). Astrocytes in chronic pain: Cellular and molecular mechanisms. Neurosci Bull.

[28] Jiang BC, Liu T, Gao YJ (2020). Chemokines in chronic pain: Cellular and molecular mechanisms and therapeutic potential. Pharmacol Ther.

[29] van Neerven S, Nemes A, Imholz P, Regen T, Denecke B, Johann S (2010). Inflammatory cytokine release of astrocytes *in vitro* is reduced by all-trans retinoic acid. J Neuroimmunol.

[30] van Neerven S, Regen T, Wolf D, Nemes A, Johann S, Beyer C (2010). Inflammatory chemokine release of astrocytes *in vitro* is reduced by all-trans retinoic acid. J Neurochem.

[31] Kampmann E, Johann S, van Neerven S, Beyer C, Mey J (2008). Anti-inflammatory effect of retinoic acid on prostaglandin synthesis in cultured cortical astrocytes. J Neurochem.

[32] Wang X, Allen C, Ballow M (2007). Retinoic acid enhances the production of IL-10 while reducing the synthesis of IL-12 and TNF-α from LPS-stimulated monocytes/macrophages. J Clin Immunol.

[33] Dheen ST, Jun Y, Yan Z, Tay SS, Ling EA (2005). Retinoic acid inhibits expression of TNF-alpha and iNOS in activated rat microglia. Glia.

[34] Zhang ZJ, Cao DL, Zhang X, Ji RR, Gao YJ (2013). Chemokine contribution to neuropathic pain: Respective induction of CXCL1 and CXCR2 in spinal cord astrocytes and neurons. Pain.

[35] Hylden JL, Wilcox GL (1980). Intrathecal morphine in mice: A new technique. Eur J Pharmacol.

[36] Jiang BC, Zhang WW, Yang T, Guo CY, Cao DL, Zhang ZJ (2018). Demethylation of G-protein-coupled receptor 151 promoter facilitates the binding of Krüppel-like factor 5 and enhances neuropathic pain after nerve injury in mice. J Neurosci.

[37] Sakhi AK, Gundersen TE, Ulven SM, Blomhoff R, Lundanes E (1998). Quantitative determination of endogenous retinoids in mouse embryos by high-performance liquid chromatography with on-line solid-phase extraction, column switching and electrochemical detection. J Chromatogr A.

[38] Jiang BC, Cao DL, Zhang X, Zhang ZJ, He LN, Li CH (2016). CXCL13 drives spinal astrocyte activation and neuropathic pain via CXCR5. J Clin Invest.

[39] Chaplan SR, Bach FW, Pogrel JW, Chung JM, Yaksh TL (1994). Quantitative assessment of tactile allodynia in the rat paw. J Neurosci Methods.

[40] Thatcher JE, Zelter A, Isoherranen N (2010). The relative importance of CYP26A1 in hepatic clearance of all-trans retinoic acid. Biochem Pharmacol.

[41] Ji RR, Berta T, Nedergaard M (2013). *Glia* and pain: Is chronic pain a gliopathy?. Pain.

[42] Zhuang ZY, Gerner P, Woolf CJ, Ji RR (2005). ERK is sequentially activated in neurons, microglia, and astrocytes by spinal nerve ligation and contributes to mechanical allodynia in this neuropathic pain model. Pain.

[43] Jin SX, Zhuang ZY, Woolf CJ, Ji RR (2003). p38 mitogen-activated protein kinase is activated after a spinal nerve ligation in spinal cord microglia and dorsal root ganglion neurons and contributes to the generation of neuropathic pain. J Neurosci.

[44] Chambon P (1996). A decade of molecular biology of retinoic acid receptors. FASEB J.

[45] Bastien J, Rochette-Egly C (2004). Nuclear retinoid receptors and the transcription of retinoid-target genes. Gene.

[46] Blomhoff R, Blomhoff HK (2006). Overview of retinoid metabolism and function. J Neurobiol.

[47] Sisignano M, Angioni C, Park CK, Meyer Dos Santos S, Jordan H, Kuzikov M (2016). Targeting CYP2J to reduce paclitaxel-induced peripheral neuropathic pain. Proc Natl Acad Sci USA.

[48] Li F, Zhu W, Gonzalez FJ (2017). Potential role of CYP1B1 in the development and treatment of metabolic diseases. Pharmacol Ther.

[49] Lim S, Alshagga M, Ong CE, Chieng JY, Pan Y (2020). Cytochrome P450 4B1 (CYP4B1) as a target in cancer treatment. Hum Exp Toxicol.

[50] Priyanka SH, Thushara AJ, Rauf AA, Indira M (2018). All trans retinoic acid attenuates markers of neuroinflammation in rat brain by modulation of SIRT1 and NFκB. Neurochem Res.

[51] Behairi N, Belkhelfa M, Rafa H, Labsi M, Deghbar N, Bouzid N (2016). All-trans retinoic acid (ATRA) prevents lipopolysaccharide-induced neuroinflammation, amyloidogenesis and memory impairment in aged rats. J Neuroimmunol.

[52] Romero-Sandoval EA, Alique M, Moreno-Manzano V, Molina C, Lucio FJ, Herrero JF (2004). The oral administration of retinoic acid enhances nociceptive withdrawal reflexes in rats with soft-tissue inflammation. Inflamm res.

[53] Hamed EA, Mohamed Farghaly HS, Abdel Mola AF, Fahmi MK, Makhlouf MM, Balfas MA (2016). Role of monocyte chemoattractant protein-1, stromal derived factor-1 and retinoic acid in pathophysiology of neuropathic pain in rats. J Basic Clin Physiol Pharmacol.

[54] Stoppie P, Borgers M, Borghgraef P, Dillen L, Goossens J, Sanz G (2000). R115866 inhibits all-trans-retinoic acid metabolism and exerts retinoidal effects in rodents. J Pharmacol Exp Ther.

[55] Verfaille CJ, Coel M, Boersma IH, Mertens J, Borgers M, Roseeuw D (2007). Oral R115866 in the treatment of moderate to severe facial acne vulgaris: An exploratory study. Br J Dermatol.

[56] Verfaille CJ, Thissen CA, Bovenschen HJ, Mertens J, Steijlen PM, van de Kerkhof PC (2007). Oral R115866 in the treatment of moderate to severe plaque-type psoriasis. J Eur Acad Dermatol Venereol.

[57] Schrage K, Koopmans G, Joosten EA, Mey J (2006). Macrophages and neurons are targets of retinoic acid signaling after spinal cord contusion injury. Eur J Neurosci.

[58] Aoto J, Nam CI, Poon MM, Ting P, Chen L (2008). Synaptic signaling by all-trans retinoic acid in homeostatic synaptic plasticity. Neuron.

[59] Chen N, Napoli JL (2008). All-trans-retinoic acid stimulates translation and induces spine formation in hippocampal neurons through a membrane-associated RARalpha. FASEB J.

[60] Stoney PN, Fragoso YD, Saeed RB, Ashton A, Goodman T, Simons C (2016). Expression of the retinoic acid catabolic enzyme CYP26B1 in the human brain to maintain signaling homeostasis. Brain Struct Funct.

[61] Zhang ZJ, Jiang BC, Gao YJ (2017). Chemokines in neuron-glial cell interaction and pathogenesis of neuropathic pain. Cell Mol Life Sci.

[62] Tsuda M, Inoue K (2016). Neuron-microglia interaction by purinergic signaling in neuropathic pain following neurodegeneration. Neuropharmacology.

[63] Zhuang ZY, Kawasaki Y, Tan PH, Wen YR, Huang J, Ji RR (2007). Role of the CX3CR1/p38 MAPK pathway in spinal microglia for the development of neuropathic pain following nerve injury-induced cleavage of fractalkine. Brain Behav Immun.

[64] Milligan ED, Zapata V, Chacur M, Schoeniger D, Biedenkapp J, O'Connor KA (2004). Evidence that exogenous and endogenous fractalkine can induce spinal nociceptive facilitation in rats. Eur J Neurosci.

[65] Nam Y, Kim JH, Kim JH, Jha MK, Jung JY, Lee MG (2016). Reversible induction of pain hypersensitivity following optogenetic stimulation of spinal astrocytes. Cell Rep.

[66] Milligan ED, Sloane EM, Langer SJ, Hughes TS, Jekich BM, Frank MG (2006). Repeated intrathecal injections of plasmid DNA encoding interleukin-10 produce prolonged reversal of neuropathic pain. Pain.

[67] Ledeboer A, Jekich BM, Sloane EM, Mahoney JH, Langer SJ, Milligan ED (2007). Intrathecal interleukin-10 gene therapy attenuates paclitaxel-induced mechanical allodynia and proinflammatory cytokine expression in dorsal root Ganglia in rats. Brain Behav Immun.

[68] Kim WM, Jeong CW, Lee SH, Kim YO, Cui JH, Yoon MH (2011). The intrathecally administered kappa-2 opioid agonist GR89696 and interleukin-10 attenuate bone cancer-induced pain through synergistic interaction. Anesth Analg.

[69] Zhou Z, Peng X, Hao S, Fink DJ, Mata M (2008). HSV-mediated transfer of interleukin-10 reduces inflammatory pain through modulation of membrane tumor necrosis factor alpha in spinal cord microglia. Gene Ther.

[70] Ma L, Peng S, Wei J, Zhao M, Ahmad KA, Chen J (2021). Spinal microglial β-endorphin signaling mediates IL-10 and exenatide-induced inhibition of synaptic plasticity in neuropathic pain. CNS Neurosci Ther.

[71] Milligan ED, Penzkover KR, Soderquist RG, Mahoney MJ. Spinal interleukin-10 therapy to treat peripheral neuropathic pain. Neuromodulation 2012, 15: 520–526;discussion526. 10.1111/j.1525-1403.2012.00462.x PMC3443506 22672183

[72] Lu Y, Jiang BC, Cao DL, Zhang ZJ, Zhang X, Ji RR (2014). TRAF6 upregulation in spinal astrocytes maintains neuropathic pain by integrating TNF-α and IL-1β signaling. Pain.

[73] Kawasaki Y, Zhang L, Cheng JK, Ji RR (2008). Cytokine mechanisms of central sensitization: Distinct and overlapping role of interleukin-1beta, interleukin-6, and tumor necrosis factor-alpha in regulating synaptic and neuronal activity in the superficial spinal cord. J Neurosci.

